# Effects of the alternative medical curriculum at the Hannover Medical School on length of study and academic success

**DOI:** 10.3205/zma001646

**Published:** 2023-09-15

**Authors:** Stefanos A. Tsikas, Volkhard Fischer

**Affiliations:** 1 Medizinische Hochschule Hannover, Studiendekanat, Bereich Evaluation & Kapazität, Hannover, Germany

**Keywords:** study success, student selection, model study course

## Abstract

**Objective:**

The model curriculum HannibaL (Hannoversche integrierter berufsorientierter und adaptiver Lehrplan) differs significantly from other medical study programs in Germany in terms of its structure with which, among other factors, the Hannover Medical School (MHH) saw an opportunity to positively influence the length of study. We investigate how the length of medical study is influenced by the curriculum’s structure and whether this has any impact on academic success.

**Methods:**

We use data from over 2,500 students who studied medicine at MHH between 2011 and 2021. We measure study time as the number of years which pass until completion of the respective study phases and academic success as the grades achieved on final exams.

**Results:**

Since they more often fail or postpone exams, students admitted based on special quotas (VQ) or a waiting list (WQ) need significantly more time to complete the first study phase (M1) compared to students who were admitted based on a selection process (AdH) or who belong to the “best school graduates” quota (AQ) because they earned the highest scores on the final secondary school exam. Yet, students from all admission groups reach the written state exam (M2) almost simultaneously. In HannibaL, WQ and VQ manage to catch up on delays from M1 with no negative impact on success in M2. In general, however, VQ and WQ achieve lower grades and drop out more often than students from AQ and AdH.

**Discussion:**

In the regular curriculum, students can only proceed with their studies once M1 has been entirely completed. HannibaL, on the other hand, allows for the catching up of delays from the first two years of study by integrating both study phases. The curricular structure thus accommodates students with lower academic performance who accumulate delays early on in their studies. By contrast, delays in the AQ and AdH groups arise during the second phase of study (M2).

## Zusammenfassung

**Zielsetzung:**

Der Modellstudiengang HannibaL (Hannoversche integrierter berufsorientierter und adaptiver Lehrplan) unterscheidet sich hinsichtlich des Studienaufbaus deutlich von anderen Medizinstudiengängen in Deutschland, unter anderem weil die MHH eine Möglichkeit sah, die Studienzeit positiv zu beeinflussen. Deshalb haben wir untersucht, auf welche Weise die Studiendauer im Medizinstudium durch die Studiengangsstruktur beeinflusst wird und ob dies Auswirkungen auf den Studienerfolg hat.

**Methodik:**

Berücksichtigt werden die Daten von über 2.500 Studierenden, die zwischen 2011 und 2021 an der Medizinischen Hochschule Hannover studiert haben. Die Studienzeit messen wir als vergangene Zeit bis zum erfolgreichen Bestehen der jeweiligen Prüfungsabschnitte, Studienerfolg über die dort erzielten Noten.

**Ergebnisse:**

Studierende der Vorabquoten (VQ) und der Wartezeit-Quote (WQ) benötigen deutlich mehr Zeit für den Abschluss des 1. Abschnitts als die Abiturbesten (AQ) und über Auswahlverfahren zugelassene Studierende (AdH), da sie häufiger Prüfungen nicht bestehen oder aufschieben. Den 2. Abschnitt des Staatsexamens erreichen jedoch alle Auswahlquoten fast zeitgleich; VQ und WQ gelingt es in HannibaL, Verzögerungen aufzuholen. Wir zeigen, dass dies keinen negativen Einfluss auf den Erfolg hat. Allgemein erreichen VQ und WQ jedoch im gesamten Studium schlechtere Noten als AQ und AdH und brechen ihr Studium zudem öfter ab.

**Diskussion:**

Im Regelstudium kann erst mit dem Studium fortgefahren werden, wenn der 1. Abschnitt komplett abgeschlossen ist. Das Aufholen von Verzögerungen aus den ersten beiden Studienjahren wird durch die Integration der beiden Studienabschnitte in HannibaL ermöglicht. Der Studienaufbau kommt also leistungsschwächeren Studierenden entgegen, die Verspätungen früh im Studium aufbauen. Verzögerungen bei AQ und AdH entstehen dagegen im 2. Abschnitt.

## 1. Introduction

Admission to medical schools is much sought after. Medical study is considered one of the most expensive courses of study in Germany and the longest undergraduate degree program, with a standard curriculum of six years. For this reason, it is important to select in advance students with the best chance of successfully completing the degree as quickly as possible. To do this, universities apply in part complex admission procedures. At the heart of these selection procedures are the qualification to study at a university (the German “Abitur”) and standardized testing (e.g., TMS, the test used for medical study programs), which have good predictive validity for study success and good grades in medical study [1], [2], [3], [4]. However, this method of selection stands in a certain opposition to the political aim of enabling all levels of society to access university education. Furthermore, the final grade achieved in secondary school is not necessarily a good predictor of practical skills or soft skills [2].

Schwibbe et al. [5] and Hampe & Kadmon [6] give excellent overviews of the selection procedures for admission to medical school and the different quota rules, which also apply to the model medical curriculum at the Hannover Medical School (MHH) [7], [8].

While admission based on the quota allotted for the best secondary school graduates (AQ) and the universities’ separate selection procedures (AdH) have not been viewed as controversial in recent years, doubts have often been cast on admissions based on the special quotas (VQ) and the waiting list quota (WQ) [9]. Studies show that WQ and VQ achieve lower grades in medical degree programs than AQ and AdH, more frequently drop out and require more time to complete M1 [7], [10]. However, how a medical program’s curricular structure concretely influences study times has largely remained unstudied.

Since 2005 MHH has offered the model curriculum HannibaL (**Hann**overscher **i**ntegrierter **b**erufsorientierter und **a**daptiver **L**ehrplan), which can be loosely translated as the Hannover integrated, professionally oriented and adaptive curriculum). This alternative medical curriculum introduces students to clinical practice from the first week onward. An important element of HannibaL is the flexibility that is built into the study program by having just one integrated phase of study instead of two strictly separate study phases, in which the second one can only be taken after the first [8]. As a consequence of integrating these two study phases and their curricular content, the subject material tested on the M1 exam is now tested consecutively in and spread out over the HannibaL modules (M1*). In addition, medical students in HannibaL may continue their studies without passing the M1*, a situation that is not possible in standard medical degree programs.

## 2. The development of regular and model curricula in Germany

### 2.1. The medical curriculum over the past 20 years

The revised version of the medical licensure regulations (ÄApprO) of April 2002 laid down a completely new system for the different parts of the state medical exam. Whereas the subjects of study remained the same in the first two years, a stronger interlinking of preclinical and clinical content was specified [11]. This meant that the amount of clinically relevant connections to be taught increased, but not necessarily the amount of clinical content. At least, this is the perspective taken in Hannover regarding the development of undergraduate medical education. The diversity of the new educational approaches is seen to some extent in a presentation of new paths in medical education [12] and in the GMA special topic issue focusing on 20 years of model curricula [13]. What is noticeable there is that the curricular structure was not indicated for most of these new approaches in Germany.

Furthermore, the group of subjects changed for the practical oral part of what is now referred to as the first part of the state medical exam (M1-new). What was formerly the first part of the medical exam, taken after the third year of study, no longer exists; the number of course attendance certificates for the clinical study phase (years 3-5) almost doubled and student performance in these courses needed to be graded. Moreover, the written exam for the clinical subjects (M2-old) was meant to be administered together with the oral exam after completion of the final practical year (M3-old). In August 2013 these revisions to the testing practices were revoked (first amendment to the ÄApprO). The prerequisite to begin the new (old) second part of the medical exam (M2-new) continued to be the graded, university-specific coursework in the clinical phase of study. Empirical studies regarding the effect of these planned changes to the curricular structure were not undertaken before the reform in 2002 nor prior to revising that legislation.

### 2.2. The model curriculum “HannibaL” at MHH

It is precisely the main concerns of the revised ÄApprO in 2002 that can already be seen in the reforming ideas of the MHH founders [14]. Following a transition period, it made sense to establish a model curriculum and to inform it based on the prior experiences gathered by the university. In particular, several contradictions between the medical licensure regulations and the capacity regulations, which set admission numbers [15], caused MHH to consciously forego having two distinct study phases in the model curriculum prior to the final practical year because that was the only way to sustainably implement the politically desired and motivationally meaningful interconnection of theoretical and clinical instruction [16].

**Figure 1:**
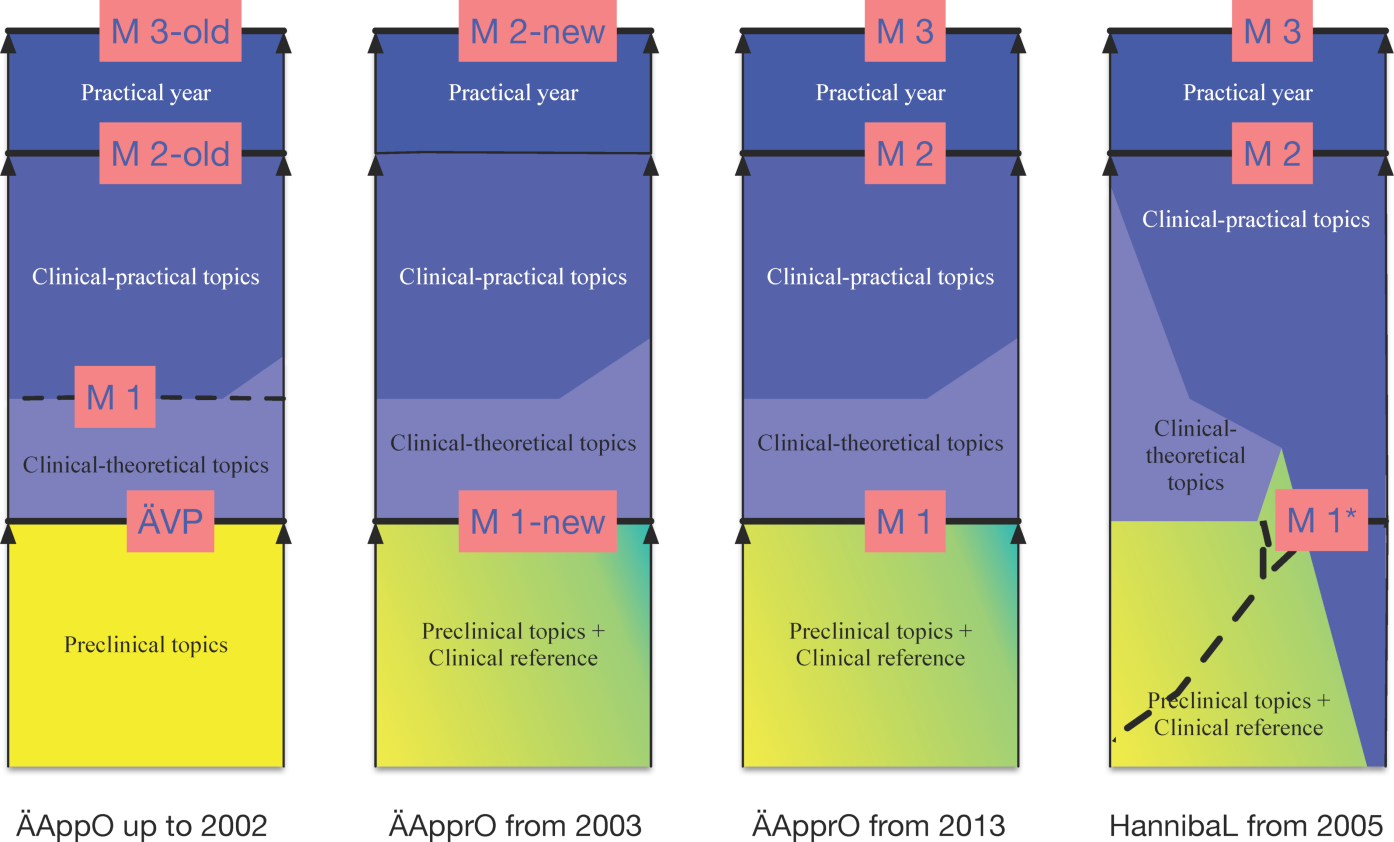
The structure of medical study in Germany according to subjects and state exams *Notes: *Left column: medical licensure regulations up to 2002 (ÄVP:= preliminary medical exam, M1:= first part of the state medical exam, M2-old:= second part of the state medical exam, M3-old:= third part of the state medical exam); middle column left of center: a regular curriculum in compliance with the medical licensure regulations between 2003 and 2013 (M1-new:= first part of the state medical exam, M2-new:= second part of the state medical exam); middle column right of center: a regular curriculum in compliance with the medical licensure regulations since 2013 (M1:= first part of the state medical exam, M2-new:= second part of the state medical exam, M3:= third part of the state medical exam; right column: the model curriculum HannibaL at MHH (M1*:= alternative exams in the model study program instead of the central M1, M2:= second part of the state medical exam, M3:= third part of the state medical exam).

Figure 1 (Fig. 1) shows the structural relationship between different curricular content and the parts of the state medical exam over the course of study in conventional medical education before 2002, in regular curricula from 2003 onward and from 2013 onward, and for the model curriculum HannibaL at MHH. As Bintaro et al. [17] have described for internal medicine, adaptations, adjustments and changes also took place with HannibaL. That aside, the intermeshing of the consecutive exams taken in the first two years of study (M1*), which are equivalent to the first part of the state medical exam, stands out as the central feature (far right column, dotted line).

### 2.3. Length of study in the regular curriculum and in HannibaL

**Figure 2:**
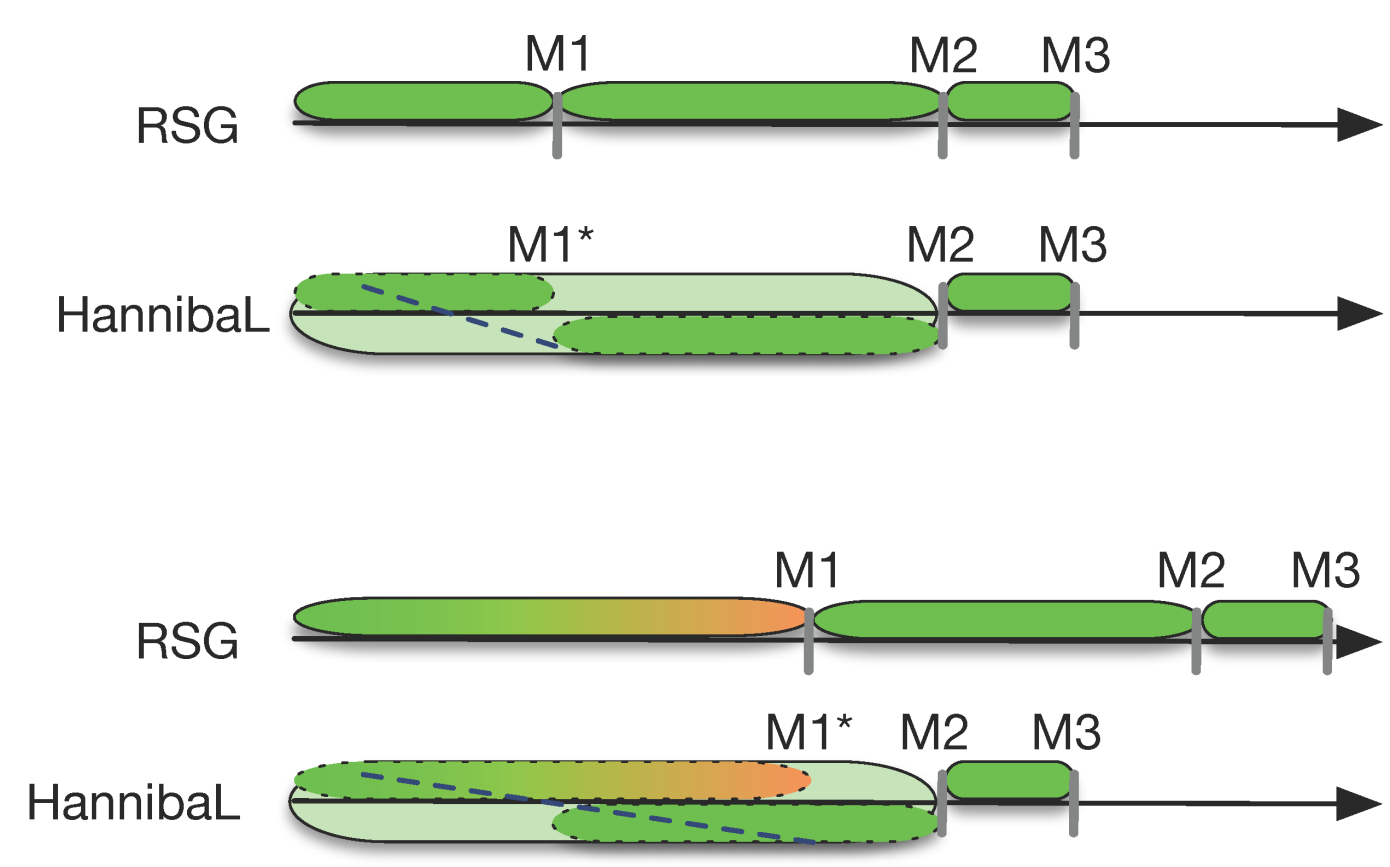
The course of study in a regular medical program (RSG) and in the model curriculum HannibaL *Notes:* Uppermost timeline: study program without delays in one of the first two study phases of the regular degree program; second upper timeline: study program without delays in the integrated study phase of the model curriculum HannibaL at MHH; third timeline: study program with a two-year-long delay in the first study phase of the regular study program; fourth timeline: study program with a two-year-long delay in the first two years of the integrated study phase of the model curriculum HannibaL at MHH.

Figure 2 (Fig. 2) illustrates this on horizontal timelines; the two upper timelines show the course of study for students in a regular curriculum and in HannibaL, who successfully pass all of the exams during the intended period of study and pass M1/M1* and M2 on the first attempt. The two lower timelines show, as examples, the course of study in a regular curriculum and in HannibaL when successfully passing M1 or completing the modules that impart basic knowledge (M1*) occurs two years later than intended by the regular curriculum.

Since the ÄApprO stipulates a minimum length of study for each study phase in the regular curricula (§ 1 subsection 3) and that students may only advance to the next phase if all of the requirements have been met for the previous phase (§10 subsections 2 and 5), any delay during the preclinical study phase will automatically lengthen the entire duration of study. In contrast, this prolongation of study need not happen in HannibaL when successful completion of M1* is delayed because the study phases are integrated, as allowed for by Section 41 (1) of the ÄApprO.

We now turn to analyzing what effects HannibaL’s flexibility has on the length of study, and if WQ students who are frequently described as academically weak [10] and some of whom have problems adjusting, especially in the beginning, to the level and tempo of medical study [7], benefit from this type of curriculum, more specifically whether or not they are able to

1. catch up on delays in completing M1* (see 4.2) so that they take the second part of the medical exam (M2) on time, and if this
2. has an influence on their passing the exam.

Analogous to [10], we operationalized the varying academic performances of the students primarily by using the admission quotas as an independent variable and not, as is done in the older studies [4], [18], by using the final grade for secondary school because our focus is on the influence of the curricular structure on academic success and not on the suitability of different admission procedures. Previous studies have shown that the final grade for secondary school has a meaningful impact on academic achievement in the first years of study and that WQ students and some VQ students can be described as clearly weaker academically than AQ und AdH students [7], [10], [19]. We show this for our sample in section 4.1 of the present study. This way of approaching the issue is also present in our study because the different admission groups are also defined based on the different admission procedures – something that particularly affects the AdH group, in that universities have great freedom when defining their selection criteria.

## 3. Data and method

For our study we use data on over 2,500 students who, between 2011 and 2021, completed the five years of integrated study in HannibaL, equivalent to the first and second study phases of a regular curriculum. A total of 13.6% of the students were admitted via the special quotas (VQ) and 16.3% from a waiting list (WQ). In our sample 12.7% belong to the best school graduates (AQ); 57.4% were successfully admitted through MHH’s own selection procedure (AdH).

To quantify the successful passing of exams during medical study, we use the grade on the written state medical exam (M2) and the final grade for the M1 equivalent: the M1* (the average grade for all written and oral module exams and for one OSCE and one elective subject). The central outcome variable here, however, is the time that elapses until M1* or M2 and connected with successfully passing the exams. In the course of doing this, we also use categorizations of those variables to differentiate between on-time completion of the (intermediary) phases from different lengths of delays.

The question if the ability to advance from M1* and M2 is associated with less delayed graduation is investigated based on descriptive group comparisons (the four admission groups) and statistical tests for significance. As with the regular curriculum, the intended length of time in HannibaL until M1* is two years, while M2 should be completed after five years of study (see figure 2 (Fig. 2)). The “normal” length of time between these two points is thus three years. This minimum duration is fixed in the regular curriculum, whereas it can be exceeded in HannibaL (e.g., delays in M1*). Therefore, a good way to answer the question is whether it is possible to make up for delays that accrue in M1*. For this reason, we address the concept of a “normal” length of study in graphic terms in section 4.2. Then, using ANOVA and correlation analysis, we also test for the influence of sociodemographic factors (namely sex, nationality, school type, and age at university enrollment) on the length of study until M1* and M2 in order to better discern the effect of the curriculum’s structure. In all of the analytical steps we identify the differences and results as being statistically significant if *p*<0.05.

## 4. Results

Table 1 (Tab. 1) presents several basic sociodemographic characteristics for our sample, according to admission group. Overall, 63% of the students are female. For WQ and VQ this percentage is clearly lower, for instance, due to specific student profiles in VQ. Foreign students are only represented in a noteworthy manner in VQ, primarily because this is the way by which students from outside the EU are admitted.

WQ students have much more frequently attended comprehensive secondary schools or other types of school compared to AQ and AdH, who usually have graduated from a conventional college-preparatory secondary school (*Gymnasium*) in Germany. Waiting times are reflected mainly in the age at university enrollment and are in part seven years higher than for the other groups.

### 4.1. Academic success

Since the length of the waiting time is the main selection criterion for WQ, the final secondary school grade is distinctly lower for WQ than for the other admission groups, as shown in table 2 (Tab. 2). The differences in the grades for M1* and M2 (except in the comparison between WQ and VQ regarding M2) are statistically significant between the admission groups (t-test). The final secondary school grade correlates significantly with M1* (*r*=0.275) and the grade for M2 (*r*=0.244). M1* has strong predictive power regarding academic performance in M2 (*r*=0.513).

VQ and in particular WQ also drop out at higher rates than AQ and AdH. Overall, the drop-out rate at MHH is lower than Kadmon et al. [10] report for the regular medical degree program in Heidelberg. AQ and AdH also reach the decision to discontinue medical study more quickly than WQ who disenroll on average after more than two years. Practically all dropouts occur without completing M1*. Transfers to another university also usually happen early on during the course of study and then pick up again before practical year. Overall, our sample confirms previous findings regarding admission groups and passing the exams.

### 4.2. Length of study and curricular structure

The length of time needed to complete M1* significantly differs between all of the admission groups at MHH (*t*-tests). Table 3 (Tab. 3) shows that students in AQ and AdH pass all of the module exams required for M1* in the intended time of two years. On average, students in WQ need almost one semester longer and VQ more than two and half years to complete M1*. In these two latter groups the outliers for a time much longer than the norm are also more prevalent.

In regard to the length of time until M2, VQ and WQ no longer differ significantly from AQ. Only the students in the AdH group need slightly less time than all of the other groups (two sample *t*-test). This applies regardless of whether M2 was administered after five years of study or after six years.

Looking at table 3 (Tab. 3), it can be seen that between M1* and M2 students in AQ and AdH exceed the usual 3-4 years for this phase by about a semester, while in VQ und WQ delays in M1* are made up for in around three years between M1* and M2 by simultaneously taking modules assigned (in the regular curriculum) to the second study phase.

**Figure 3:**
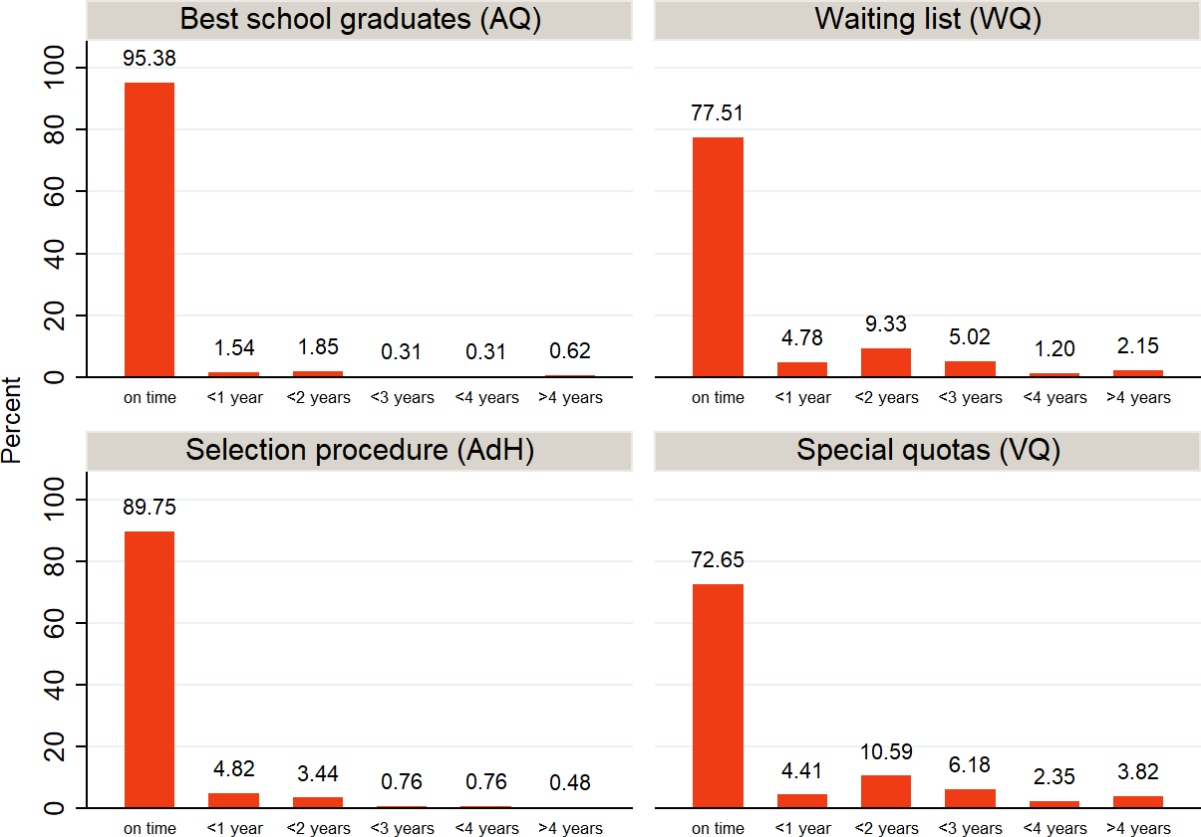
How many students complete M1* in the amount of time intended by the regular curriculum? *Note:* M1 equivalence was defined as “punctual” if the length of study time did not exceed 2.2 years.

Figure 3 (Fig. 3) shows that over 95% of AQ and almost 90% of AdH complete M1* within two years. For those who were admitted to MHH based on a waiting list or special quota, this percentage falls to 77.5% and 73%, respectively. In the two latter groups, approximately 95% complete M1* in a length of study time under five years; in each group almost 5% needed 1-2 semesters more for M1* and another 10% exceeded the intended two years of study by 3-4 semesters.

**Figure 4:**
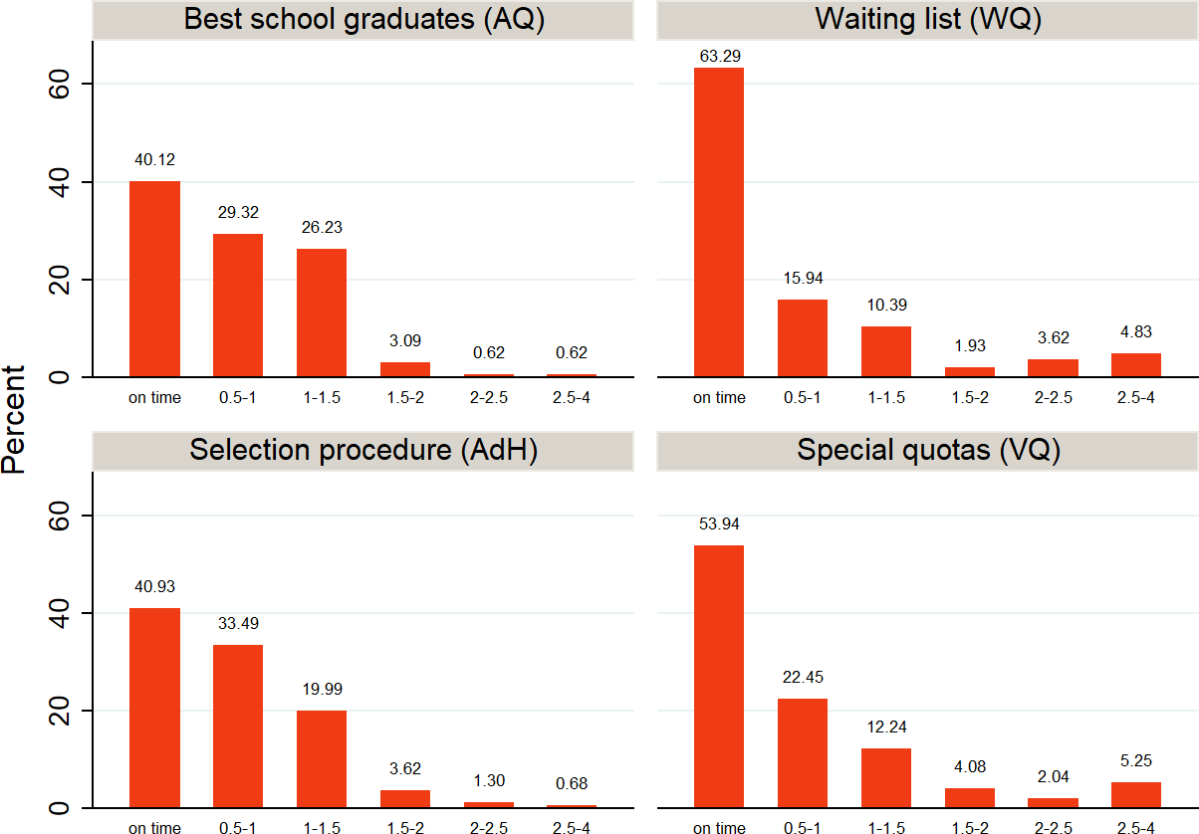
How many students complete M2 in the amount of time intended by the regular curriculum? *Note: *M2 was defined as “punctual” if the length of study time before passing the M2 exam was less than 5.5 years or 6.5 years (up to an including 2014).

Figure 4 (Fig. 4) shows that across all admission groups distinctly fewer students keep to the “normal” amount of three years (formerly four) between M1 and M2. In all of the groups students are exceeding the intended study time to reach M2 (five years) by somewhat more than one semester (see table 3 (Tab. 3)). In AQ and AdH around 5% exceed the “normal” duration of study by more than 1.5 years; for the two other groups, this amount is more than double that. Yet, with 63% (WQ) and 54% (VQ) considerably more students complete the M2 exam on time.

**Figure 5:**
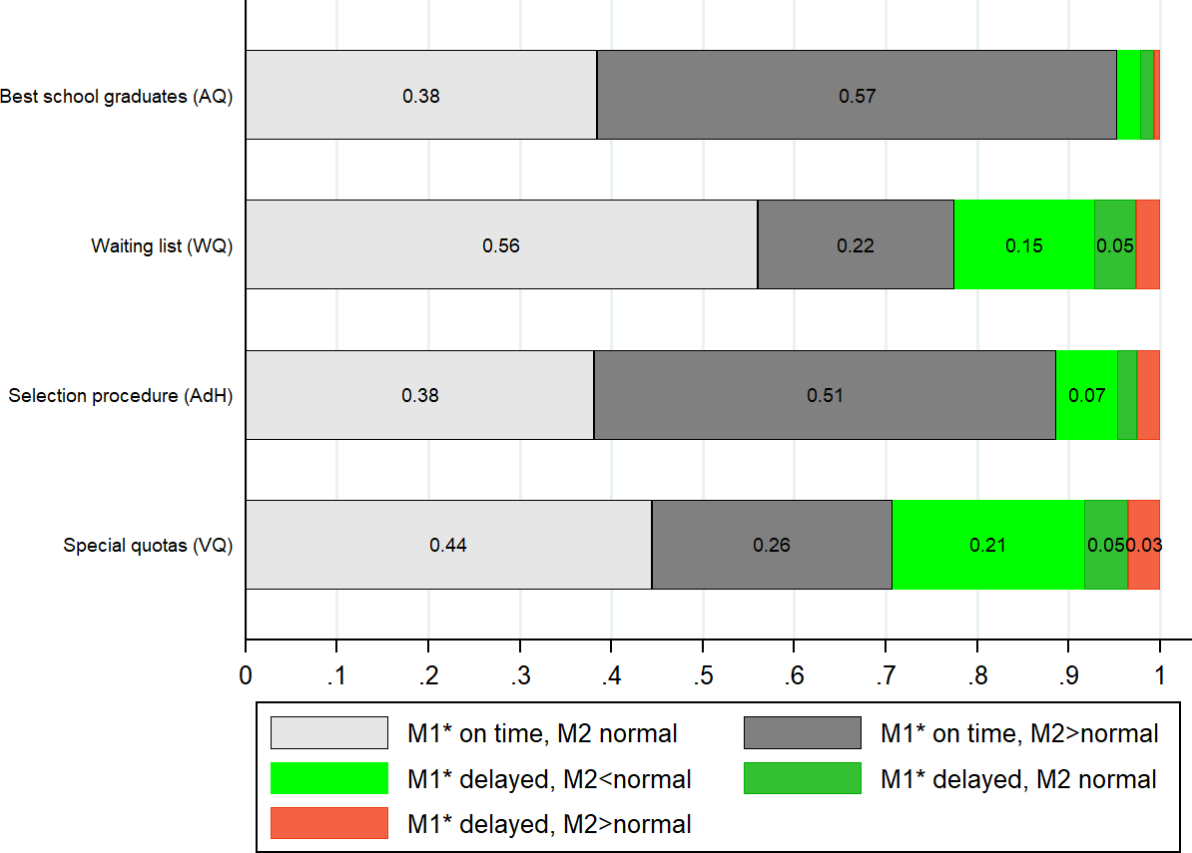
Study time for M2 depending on delay in M1* *Note: *M1* was completed on time if the length of time did not exceed 2.2 years; “normal” describes a time period between M1* and the M2 exam under 3.5 years (4.5 years if M2 and M3 were tested together after the final practical year of medical study). All values given as a percentage of 1.

Figure 5 (Fig. 5) illustrates how the length of time needed for M1* and M2 are connected to each other in HannibaL. While almost all AQ reach M1* after two years, 62% need more than five years to reach M2. Similar findings are seen for the AdH group. Only the percentage of students who, after delays in M1*, need less than the “normal” study time is somewhat higher.

Figure 5 (Fig. 5) makes clear that, following a delay in M1*, 15% of WQ and 21% of VQ (green bars) take the M2 exam on time after five years and potential delays from the first years of study can be compensated for (analogous to this, the percentage of students in this group who exceed the “normal” amount of time is smaller). As described above, this cannot happen in the regular medical study programs. Figures A1 and A2 in attachment 1 illustrate that individual students from WQ are successful in (very nearly) fully compensating for delays. However, the greater the amount of time needed to complete M1*, the less often this is possible.

### 4.3. Length of study and academic success

Table 4 (Tab. 4) shows that students who reach M1* late, demonstrate across all admissions groups significantly (t-test) poorer performance than those who reach it within the intended amount of time. In the subgroup of students with delays, we find no statistically significant differences between the four admission groups.

In the subgroup of students who are punctual, AQ stands out with an average grade of 1.6. The other groups are relatively close to each other; what is mainly noticeable is that the grade on the M1* achieved by VQ does not differ statistically from AdH.

Within VQ it is important where the students come from: 26% of VQ students attained their qualification to study at a university in a non-EU state. These students require almost three and a half years for the first study phase (because exams are more often failed or postponed), while their German counterparts in VQ come close to those in AQ with a length of study time of 2.2 years. The average M1* grade for all German students is almost exactly the same grade achieved by the punctual VQ students. A punctual M1* is positively associated with the M2 grade, regardless of whether or not the “normal” length of study was exceeded between M1* and M2. For all of the groups a poorer performance on the M2 follows an M1* delay. This is independent of whether or not the “normal” amount of time is exceeded between M1* and M2. A detailed illustration of these results is presented in Table A3 in attachment 1 .

### 4.4. Sociodemographic factors and length of study

Table 5 (Tab. 5) shows the results of our ANOVA and correlation analyses regarding the influence of sociodemographic characteristics on the length of study time until M1* or M2: German students reach M1* significantly more quickly than foreign students. We were unable to find an effect for M2. Attaining the qualification to study at a university from a German *Gymnasium* (college-preparatory secondary school) is also clearly associated with completing M1* on time. This influence remains significant for the length of study time needed to reach M2; however, the F statistic decreases considerably.

The final secondary school grade, age and M1* correlate moderately with the length of time for M1*, but not with the time until M2. Only a higher M1* grade is connected with more time required to reach M2. Place of origin and type of school (in addition to the final school-leaving grade) also have the greatest influence on the grades in M1* and M2, whereby it is less pronounced for the M2 outcome than for M1* (not shown in table).

## 5. Discussion

Especially in the regular medical study programs, students who have been admitted based on special quotas or from a waiting list not only earn lower grades and drop out more frequently, but also generally take longer to become licensed physicians [10]. Delays in medical study usually come about in the first two years of study. This phase, which covers basic scientific knowledge, is in terms of the topics still rather like secondary school instruction, even though it is definitively more complex and taught at a faster tempo. It is not surprising that it is primarily a student’s final secondary school grade (and thus the admission group) that explains the loss of time during M1*. For WQ students, it is also the case that several years have passed since secondary school graduation and any learning strategies connected with it, which can make it more difficult to embark upon higher education. Another factor in our study that favors a delayed completion of M1* is place of origin. We argue that language barriers and integration into a different culture/social environment are explanations [20], [21] and find that foreign students habituate themselves quite quickly and catch up to WQ students. German students falling under the heterogenous special quotas, e.g., prospective military doctors or those pursuing a second university degree, are very close to the AQ and AdH from the start in terms of their academic performance and length of study time, which can also be explained based on their grades on the final secondary school exam, which fall into the (lower) range for that of the AdH students.

Depending on cognitive abilities, origin, path of life or personal reasons, some students will not be able to smoothly complete the degree program or adjust as easily to the demanding pace of medical study. With the revised structure of HannibaL, MHH has taken this fact into account by relaxing the strict division between the first and second study phases. Students who do not complete all of the modules required for M1* within the first two years of study can still begin the clinically oriented phase in years 3-5 and take or retake exams if they feel ready. Passing the OSCE in the Diagnostic Methods module (second year of study) is the prerequisite for beginning the clinical clerkships, not M1*.

When looking at the length of study time that elapses before M2 is passed, we no longer see any differences between the admission groups. Our analysis provides the following explanations:

- WQ and VQ students take more exams in years 3-5 than AQ students because they are catching up on coursework during this time. A delay in M1* which can, in part, be compensated for in this manner (see figure 5 (Fig. 5)) has no negative effect on the M2 grade.
- Failed exams at the beginning of medical study are common for WQ and foreign students. In years 3-5, failed tests are rare in all of the admission groups. This means that students have acclimated themselves to the demands of medical study and, thanks to HannibaL, have the opportunity to make up for delays originating in M1* without having to postpone the next study phase.
- All of the admission groups exceeded the amount of time allotted for M2 by somewhat more than one semester. While low academic performance leads to a delay in M1*, delays are seen for the AQ and AdH groups later in the course of study.
- It is to be assumed that delays after M1* and before M2 are mainly due to plans to pursue a doctorate. The data available on this are not sufficiently specific to quantify the effect of writing a dissertation on the length of study. Based on internal MHH surveys and data collection, we know that AQ (90%) and AdH (70%) more frequently begin pursuing a doctorate than WQ and VQ (nearly 60% for each group, although self-reported information need not be representative).
- Since delays between M1* and M2 are also influenced by factors which are not associated with cognitive abilities or are even positively associated with them (doctoral degree), the length of study time until M2 (in contrast to M1/M1*) is not a valid predictor of academic success.
- It is possible that students with prior work experience (primarily WQ) more quickly pursue the practice of medicine than their peers in the AQ and AdH groups. This has not yet been empirically investigated. In addition to possible reasons such as previous work experience or personal preferences, our results suggest that the accumulated delays and (in conjunction with starting the study of medicine at an older age) certain financial constraints also play a role.

Despite higher rates for delays and dropping out during the first years of study and lower grades, most of the WQ and VQ students also receive medical licensure. This is good news given the advancing demographic shift with potential shortages in healthcare and the political goal of making access to medical study more socially diverse – especially because WQ students (as of very recently defined as a quota for the professionally experienced (BEQ)) bring with them practical skills that are often and rightly identified as important. In the present study we have demonstrated that such students, despite poorer school leaving grades, are able to adjust and that adding flexibility to the curricular structure can help students with lower academic performance make up for deficits and delays during the course of study which, in turn, enables a (timelier) start in the medical profession. Several studies found (in the case of an “advantageous” curriculum) a positive association between practical experiences and study success [22], [23]. Alongside the goal of strengthening practical medical competencies during medical study, prior work experience has also recently gained importance in the process of selecting students. Previous medical experience can be a bonus for both the group of students with special aptitudes and the AdH group, often in connection with the final grade in secondary school (but not for the special aptitude group) or academic aptitude tests as cognitive components.

There is virtually no systematically gathered data on curricular structure and study duration for other medical schools (with regular or model curricula), which is why there are no comparative values for the advantages of the MHH model curriculum demonstrated here. In particular, we do not know when, how, why or to what extent delays happen before M1 in regular medical study programs. Although Zimmermann et al. and van den Bussche et al. [24], [25], [26] present extensive analyses of academic success in different study phases, it is based on data which cannot represent the options and possibilities arising from structural differences. In terms of future research, a multicentric study comparing model and regular medical study programs would be of great interest. In this respect, we view our study as a pilot and as the first step in the direction of further joint studies. This also applies to medical school graduates’ decisions regarding careers and post-licensure education and training. We have made several assumptions in our study based on the results and which we are not able to verify. It is precisely in view of the looming shortage of physicians in rural areas that investigating which types of students tend toward general practice is of interest [27].

## Competing interests

The authors declare that they have no competing interests. 

## Supplementary Material

Supplementary material

## Figures and Tables

**Table 1:**
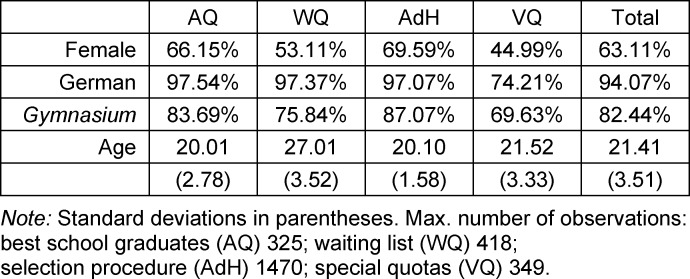
Sociodemographic variables

**Table 2:**
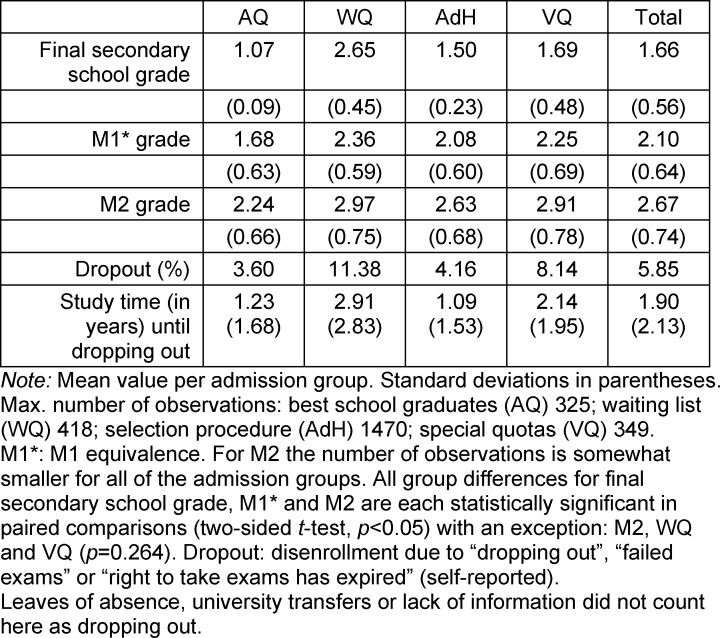
Exam success per admission group

**Table 3:**
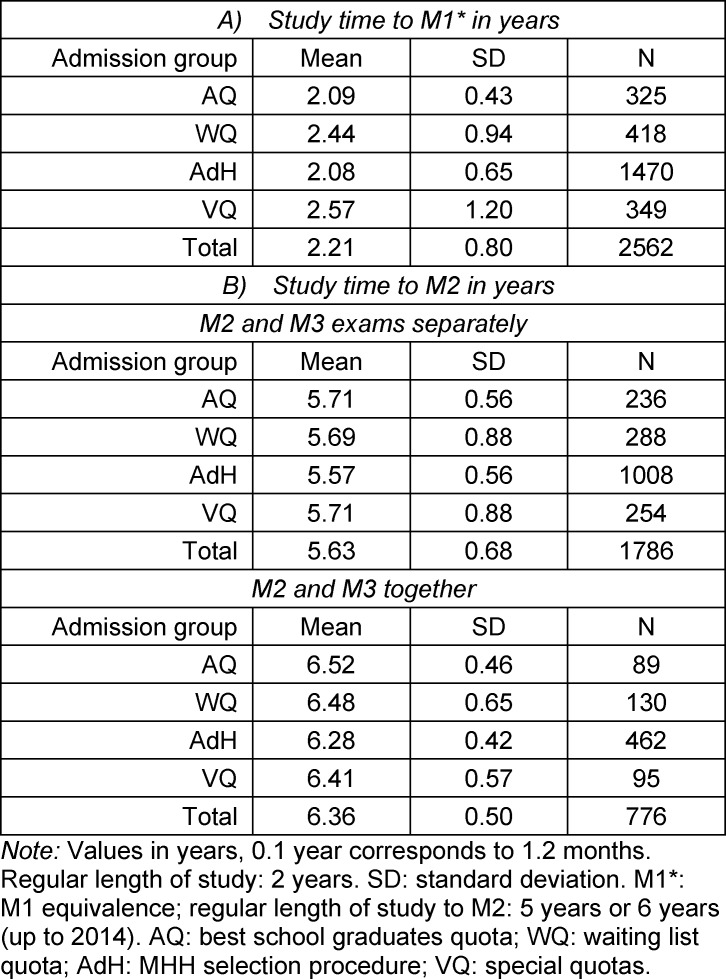
Study time to M1* and M2

**Table 4:**
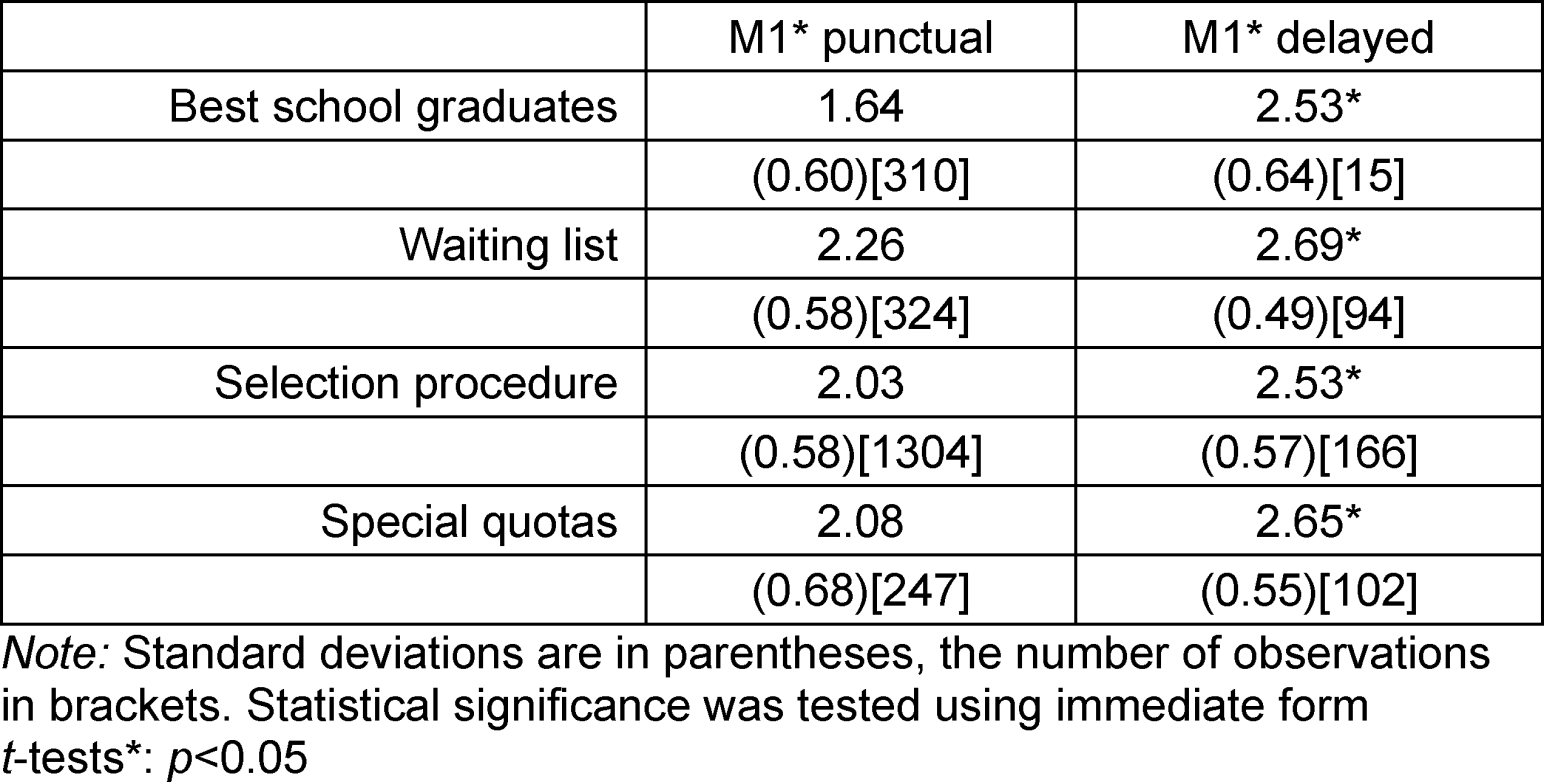
Normal length of study and M1*

**Table 5:**
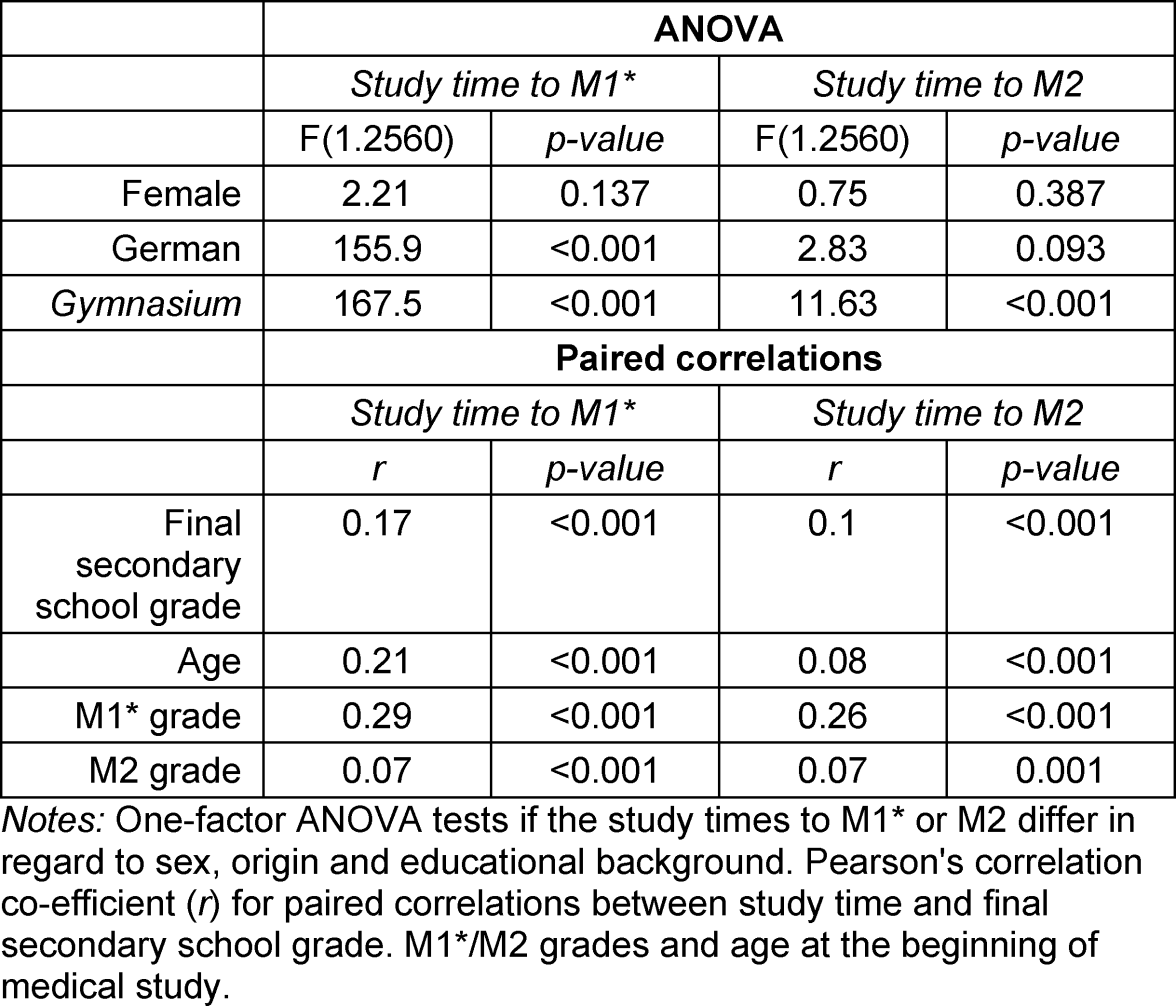
Sociodemographic characteristics and length of study
