# Supplementary material for: Effects of the alternative medical curriculum at the Hannover Medical School on length of study and academic success
Source: GMS J Med Educ. 2023 Sep 15;40(5):Doc64. doi: 10.3205/zma001646 (PMC10594034; doi:10.3205/zma001646)
Supplement: Supplementary material [file JME-40-64-s-001.pdf]

Attachment 1: Supplementary material

Figure A1: Exceeding the length of time intended for M1* and length of time between M1* and M2, for students admitted via the waiting list (WQ)

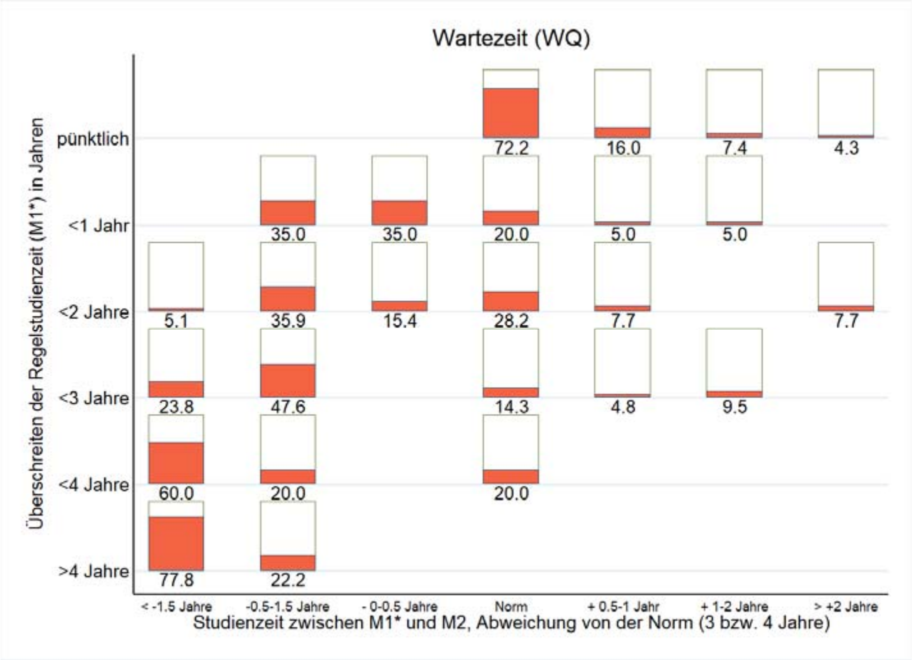

Note: Length of study time between M1* and M2 (ordinate) depending on the delay in M1*. The red bars indicate the percentage of students per category of M1* delay. If M1* (after two years) and M2 (generally in five years) are both completed "on time," then the length of study time from M1* to M2 is three years. For instance, almost 80% of WQ students who completed M1* after more than three but less than four years kept within the normal length of study time or even finished sooner.

Figure A2: Exceeding the length of study intended for M1* and length of study between M1* and M2, for students admitted based on special quotas (VQ)

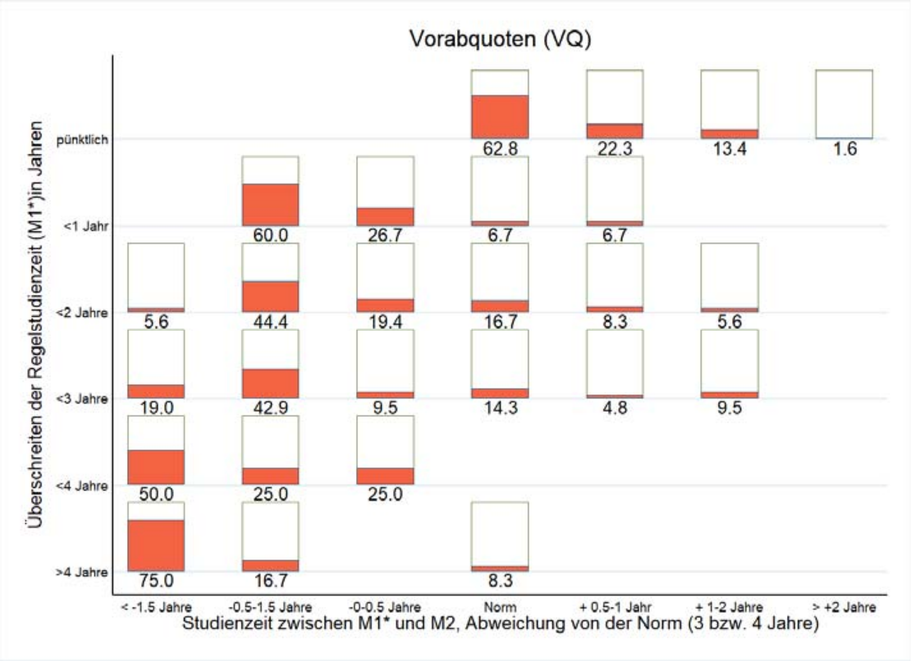

Note: Length of study time between M1* and M2 (ordinate) depending on the delay in M1*. The red bars indicate the percentage of students per category of M1* delay. If M1* (after two years) and M2 (generally in five years) are both completed "on time," then the length of study time from M1* to M2 is three years.

Table A3: M2 grades according to length of study time for M1* and M2

	(1) M1* delayed & M2 ≤ norm	(2) M1* delayed & M2 ≥ norm	(3) M1* punctual & M2 ≥ norm
Best school graduates	2.91	2.86	2.21
	(0.70)[11]	(0.90)[7]	(0.64)[281]
Waiting list	3.36	3.26	2.86
	(0.69)[75]	(0.76)[27]	(0.73)[299]
Selection procedure	2.98	2.84	2.59
	(0.67)[117]	(0.67)[64]	(0.67)[1204]
Special quotas	3.39	3.11	2.72
	(0.67)[79]	(0.88)[28]	(0.71)[228]

Note: "Norm" indicates a length of study time between M1* and passing the M2 exam of under 3.5 years. M1* was punctually completed if the length of study time did not exceed 2.2 years. Standard deviations in parentheses; the number of observations in brackets.
